# An integrated expert weight determination method for design concept evaluation

**DOI:** 10.1038/s41598-022-10333-6

**Published:** 2022-04-15

**Authors:** Zhe Chen, Peisi Zhong, Mei Liu, Qing Ma, Guangyao Si

**Affiliations:** 1 Advanced Manufacturing Technology Center, Shandong University of Science and Technology, Qingdao, 266590 China; 2 College of Mechanical and Electronic Engineering, Shandong University of Science and Technology, Qingdao, 266590 China; 3 Shandong Jiaotong University, Jinan, 250357 China; 4 Yantai Research Institute and Graduate School of Harbin Engineering University, Yantai, 264006 China; 5 School of Minerals and Energy Resources Engineering, University of New South Wales, Sydney, 2052 Australia

**Keywords:** Applied mathematics, Information technology

## Abstract

Expert weight determination is a critical issue in the design concept evaluation process, especially for complex products. However, this phase is often ignored by most decision makers. For the evaluation of complex product design concepts, experts are selected by clusters with different backgrounds. This work proposes a novel integrated two-layer method to determine expert weight under these circumstances. In the first layer, a hybrid model integrated by the entropy weight model and the Multiplicative analytical hierarchy process method is presented. In the second layer, a minimized variance model is applied to reach a consensus. Then the final expert weight is determined by the results of both layers. A real-life example of cruise ship cabin design evaluation is implemented to demonstrate the proposed expert weight determination method. To analyze the feasibility of the proposed method, weight determination with and without using experts is compared. The result shows the expert weight determination method is an effective approach to improve the accuracy of design concept evaluation.

## Introduction

Design concept evaluation is a critical phase in new product development (NPD). An ideal initial design concept can match customers’ requirements, and save time and cost for companies in the competitive global market^1^. The preliminary design concept often shows the novelty, feasibility and quality of the product. Design concept evaluation in the early stage is a phase to choose a suitable plan from the initial design concepts. It is essential in the early stage of NPD, especially for complex products^2^, because once the design concept is fixed, it is not easy to modify it in later stages. The requirements and preferences of customers and the structure and material of the product are determined in this phase^3^. The cost and sustainability of the product are also estimated.

**Figure 1:**
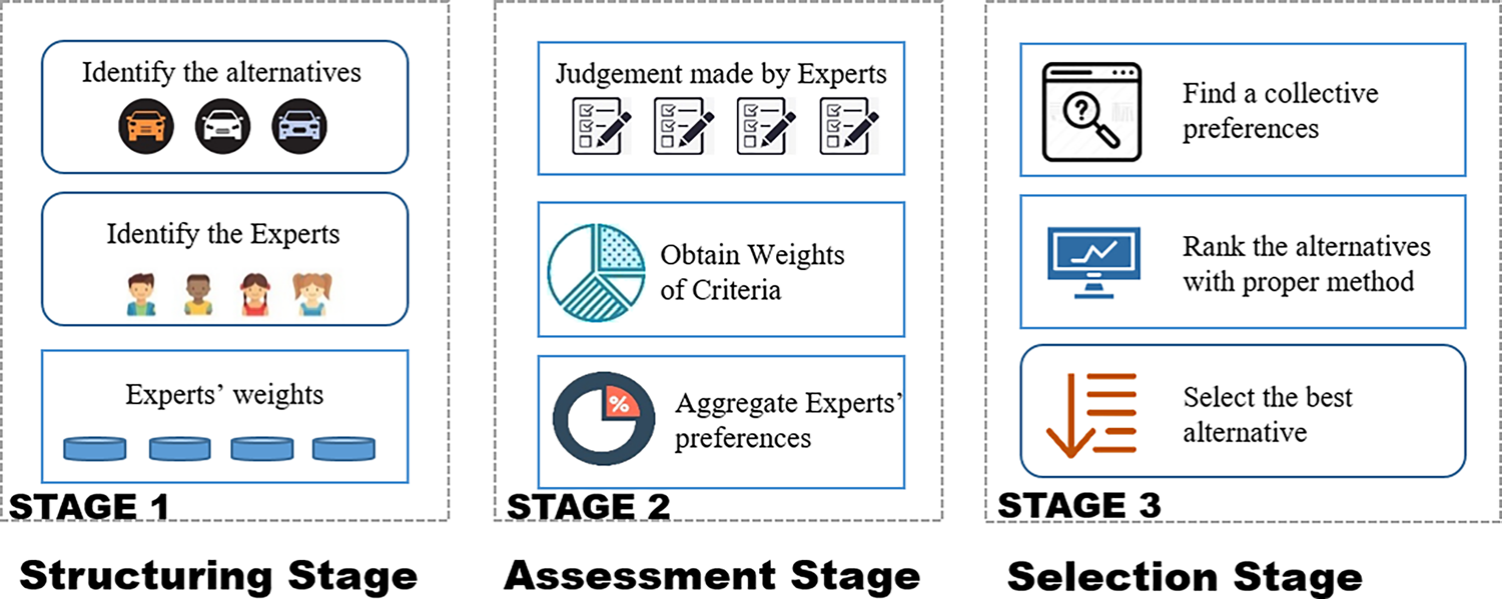
Design concept evaluation process.

As the design concept evaluation is based on a variety of factors, it can be seen as a multiple attributes decision making (MADM) problem. In MADM problems, due to the uncertain environment, individual judgement may be imprecise and subjective, and thus group decision making (GDM) is an effective solution in design concept evaluation^4^. In GDM, the ranking of the alternatives is recommended by integrating experts’ judgements. Kabak et al.^5^ reviewed related literature and proposed a generic conceptual MADM framework with three stages, as shown in Fig. 1. The first stage is the structuring stage. Alternatives, attributes and experts are defined in this stage, and the weights of experts are also determined here. After that, in the assessment stage, the weights of attributes are obtained. In the final selection phase, the alternatives are ranked based on an appropriate mathematical model.

In the past, the alternative ranking approach attracted the most significant attention. Up to 2010, over 70 selection methods had been previously proposed in MADM problems. According to King^6^, the analytical hierarchy process (AHP), utility theory, graphical tools, quality function deployment (QFD) and fuzzy logic^7,8^ approaches are the most popular methods in design concept evaluation. In particular, the Technique for Order Preference by Similarity to Ideal Solution (TOPSIS), the VlseKriterijumska Optimizacija I KOmpromisno Resenje (VIKOR), the ELimination Et Choice Translating REality (ELECTRE) methods and their extended approaches have been developed to address MADM problems^9–12^. Scholars are willing to establish a proper evaluation model to deal with specific real-life projects. However, studies on expert weight determination are very limited^13,14^. Expert weight determination is only mentioned in 41% of the top-cited papers on GDM. Therefore, experts are treated as a homogeneous group by default, and the individual weight is set to average. Design concept evaluation is a specific application area of MADM methods^1^, and the most frequently cited studies in this area are listed in Table 1. Although researchers have made efforts to optimize the method to obtain criteria weights, expert weight is rarely mentioned.

**Table 1:** Highest cited studies in design concept evaluation.

Reference (year)	Journal	Expert weight determination method	Criteria weight determination method	Example
Zhai (2009)^15^	Expert Systems With Application	N/A	Grey relation and rough set	Illustrative example
Zhu (2015)^16^	Advanced Engineering Informatics	N/A	Rough number based AHP	Lithography tool
Tiwari (2016)^17^	Advanced Engineering Informatics	N/A	Rough set and VIKOR	Testing ring machine
Geng (2010)^4^	Expert Systems With Application	N/A	Vague cross-entropy method	HDD machine
Song (2013)^1^	Journal of Engineering Design	N/A	Rough number-based AHP	Mini-fridge
Shidpour (2016)^18^	Expert Systems with Applications	N/A	Extendede method on fuzzy AHP	Mobile
Zhu (2020)^19^	Applied Soft Computing	N/A	Fuzzy rough-number based AHP	Heat exchanger

**Figure 2:**
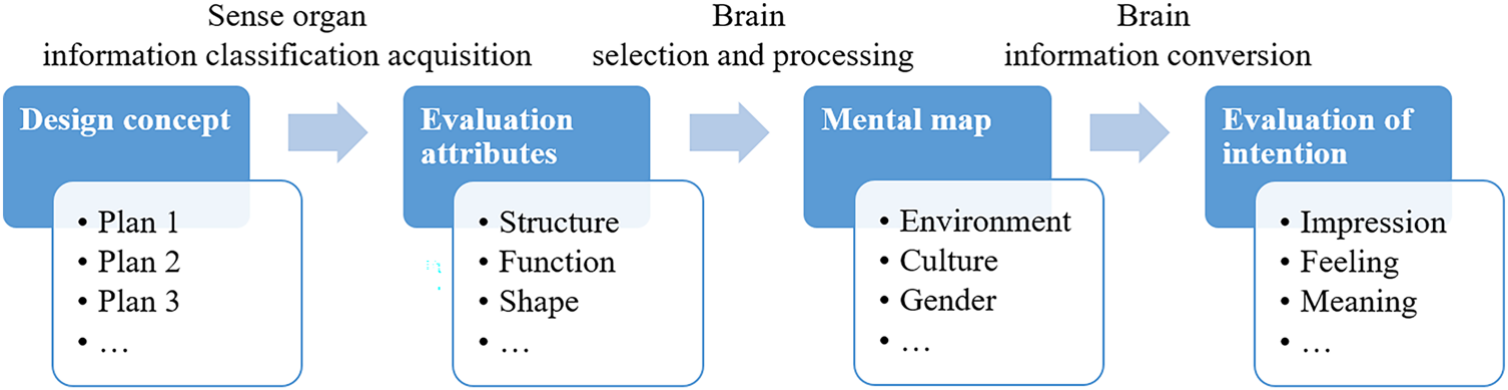
Cognitive system of design concept evaluation^20^.

In Fig. 2, from the cognitive system of design concept evaluations, we can infer that an expert needs to make an individual judgement based on their cognition and preference. Thus, the judgement is subjective, depending on the expert’s knowledge, culture, experience and aesthetic. However, it is practically impossible to form a group of experts with a similar background. The drawback of an individual in the expert group may influence the accuracy of experts’ judgement.

Once the weight of each expert is distributed equally or ignored, the final decision may lead to an incorrect result. Thus, some researchers started to develop an appropriate method to determine the expert weight. In their investigations, not every selected expert is familiar with all the criteria requiring judgement, and some specialized criteria may be beyond the cognition of some experts. Thus, the contribution of experts may not be equal in the decision process. It is essential to determine the weight of experts in the decision process to eliminate any deviation caused by the experts’ imprecise cognition.

**Figure 3:**
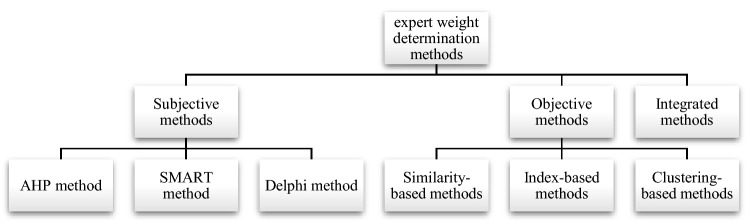
Classification of weight determination methods.

Generally, there are three ways to solve weight determination problems: the subjective approach, the objective approach and the integrated approach, as shown in Fig. 3. For the subjective methods, the weight of experts is calculated by integrating the evaluation of each other expert, depending on their age, attitude, experience etc.^21^. The objective methods are based on available evaluation data, and no extra information is required. Hence, the expert weight is computed based on an appropriate mathematical model^22^. Compared to the subjective methods, they are generally more objective, but they ignore the expert’s personal preference. In this part, the expert weight determination methods are reviewed.

In the early stage, subjective methods dominated in studies on expert weight determination. Expert weights were assigned by the supervisors or by comparison between individual expert groups. In the subjective expert weight determination, pairwise comparison matrices were established, then methods with a geometric mean were proposed to determine the expert weight. Multiplicative AHP^23^ and Simple Multi-Attribute Rating Technique (SMART) are two key approaches in subjective expert weight determination^24^.

Ramanathan and Ganesh^23^ were the first scholars to develop the Multiplicative AHP framework in the expert weight determination area. They proposed an eigenvector based method by comparing the experts’ influence in pairs, as interpersonal comparison can help experts achieve a consensus without any interaction^23^. Barzilai et al.^24^ improved the Multiplicative AHP method and explained the relationship between the Multiplicative AHP method and the SMART method. The geometric mean of the scaled gradation indices or values is applied in both methods^21^. As well as the methods mentioned above, Delphi is another competent subjective approach in expert weight determination. Azadfallah^25^ applied the Delphi technique by comparing the attributes by experts in pairs, then computed the weights with the eigenvector method.

Subjective expert weight determination methods are based on relative judgements. To make precise judgement, the experts are expected to be familiar with every attribute. However, in real-life projects, it is not practical for every expert to meet all the requirements. Moreover, once the number of attributes, alternatives, or experts increases, pairwise comparative work would be a huge project. Due to the heavy workload, pairwise comparative judgements are not easy to implement in complex MADM problems. Objective weight determination methods have developed rapidly in recent years. There are three objective expert weight determination methods, as well as some special methods such as the Markov Chain’s theory^26^.

In similarity-based methods, expert weights are determined by distinguishing the expert’s evaluation or measuring the expert’s distance to the aggregated decision^27,28^. The similarity-based study is very close to the TOPSIS method, in which the expert whose decision has minimum distance to the ideal solution has the highest weight. Yue^28,29^ proposed a modified TOPSIS method, to determine the expert weights by measuring the distance between the expert judgement and the ideal decision. Yang proposed a rough group decision matrix to determine the expert weights^30^. Wan^31^ constructed a bi-objective program which could maximize the minimum degree of acceptance and minimize the maximal degree of rejection, and also introduced three approaches to measure the distance between the individual preference and the group. Jiang^32^ introduced a novel method to measure the distance between the expert preference and the ideal solution.

Index-based methods are divided into two groups: consensus-based methods and consistency-based methods. In consensus-based methods, experts are assigned to adjust their preferences, or vary their weights to make their judgements more similar^33^. Pang^34^ developed a non-linear programming model, and determined the expert weight by maximizing the group consensus where the expert weight adjusts adaptively with the experts’ decision. Xu^35^ also used a non-linear model, and proposed a genetic algorithm in expert weight determination. Dong^36^ introduced a consensus reaching process in group decision making. The method is proposed for non-cooperative expert groups, and the expert weights are determined dynamically by a self-management mechanism. Consistency-based methods are superior in inaccurate judgements, using consistency indexes against the group decision to determine the weights of experts^37^. When some experts have higher reliabilities compared to others, the decision makers need to reach a consistent view through negotiations. Liu^38^ introduced an expert weight adjusting method based on the consistency-based method. The expert weights are firstly computed by the AHP method and the entropy method, and then optimized by the consistency level of a black-start result. Chen^39^ established a collective consistency matrix of all experts and determined the weight of experts by the consistency degree of each expert.

**Figure 4:**
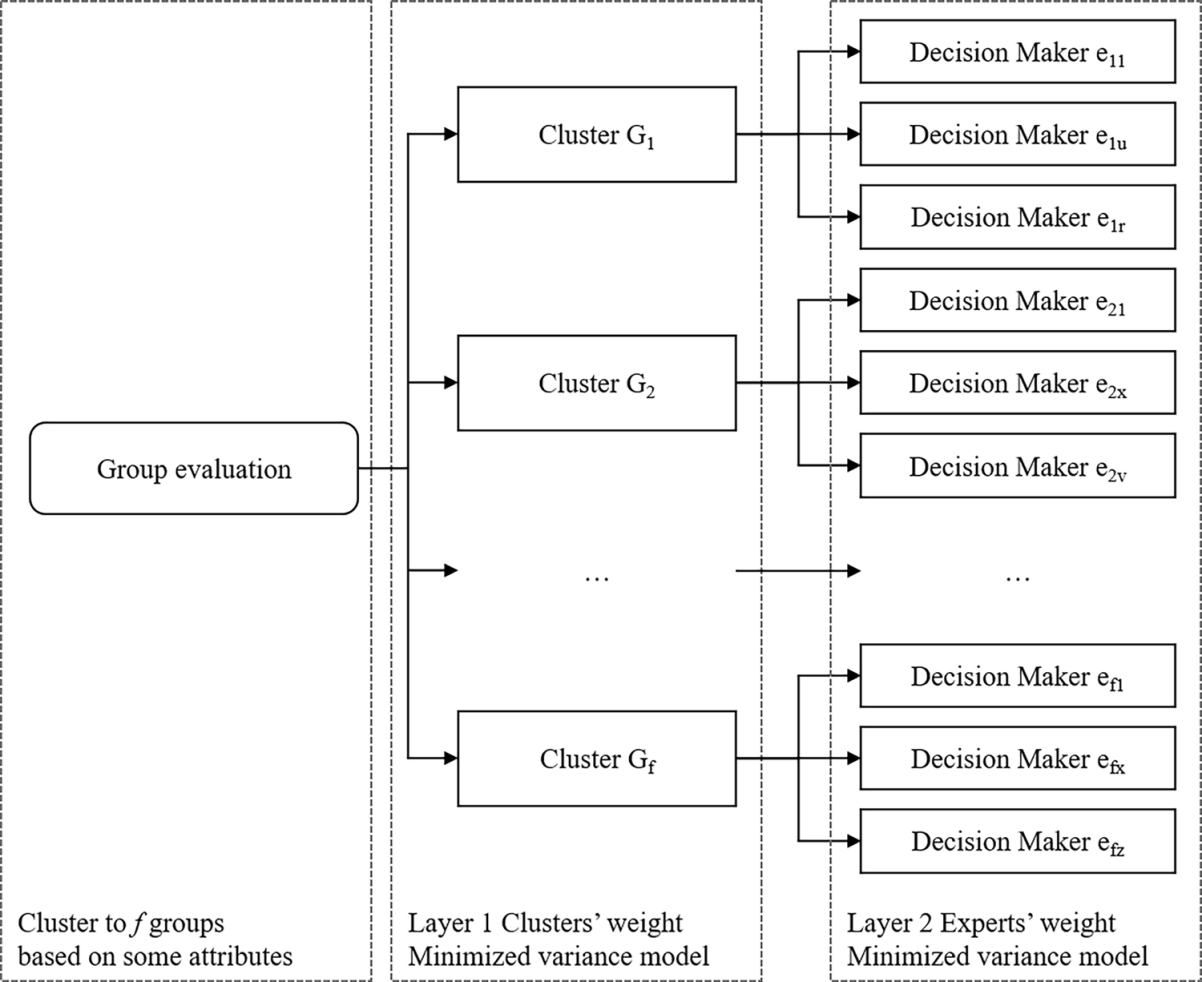
Two-layer expert weight determination method by Liu^40^.

When the group is large, a clustering-based method may be appropriate in real-life problems. A multi-level weight determination method may optimize weight via different models according to the features of the current layer^40^. Sometimes experts are assembled in several specific groups, and the experts in the same group have a similar background, but the gaps between groups are large. As shown in Fig. 4 by Liu^40^, a two-layer model is proposed based on the 2-tuple linguistic (2TL) model. In the first layer, decision makers are separated into clusters by certain criteria. The weight of the group depends on the importance of itself. The clusters are regarded as small systems with similar individual information, which is a well-organized system. Liu utilized an Entropy Weight Model to reach consistency. In each cluster, the experts’ status, occupations and experiences are close. Therefore, their judgements should be similar but not identical^41^. To reach a consensus, a Minimized Variance Model is implemented in this layer to seek a minimized deviation among all variables.

The literature proposed problem-solving methods through a specific mathematical model or operator to determine expert weights, through methods such as comparing the deviation between the expert preference and the ideal solution, or simply adjusting the expert weight to make the experts achieve consensus. However, there are still some drawbacks. First, as some of the mathematical models or operators may focus on the overall average, some information that deviates from the average level may be ignored. Second, existing studies usually obtain the expert weight by a specific method. Once the problem is complex, one single method cannot reflect a real-life problem accurately.

The integrated method relies on an integration of two or more methods to eliminate the drawbacks of a single approach or simplify a complex method. As integrated methods show their advantage in solving complex GDM problems which have become more pronounced in recent years, an increasing number of integrated expert weight determination methods are presented in decision making^22^. Qi^42^ proposed models based on various conditions under interval-valued intuitive fuzzy decision environments and determined both criteria weight and expert weight. First, they introduced a method to measure the gap between decision matrices and the ideal decision matrix, and then they developed an approach to evaluate the similarity degree between individual decision matrices. Liu^43^ proposed another expert weight determination method. The method integrated both subjective and objective expert weight determinations in decision making. First, a plant growth simulation algorithm is applied to get the generalized Fermat–Torricelli point of individual preferences with interval number decision matrices. Then a similarity-based expert weight determination method is used. Finally, the expert weights obtained from both methods are aggregated. Jabeur^44^ also determined the expert weight based on subjective and objective components.

**Figure 5:**
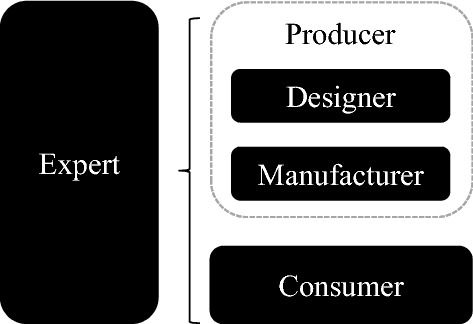
Development of design concept evaluation experts.

In product design concept evaluation, a group of experts must be selected in a proper way to ensure the correctness of the assessment. In complex product design concept evaluation projects, as shown in Fig. 5, experts are normally selected from experienced consumers and expert producers^45^.

Normally, most expert producers are designers and manufacturers. Hence, we can categorize the decision makers into the designer cluster (DC), the manufacturer cluster (MC), and the consumer cluster (CC). The cluster category can perfectly reflect the clustering information of experts. In addition, the integration of subjective and objective weights can improve the accuracy of the design concept evaluation^46^. However, recent studies seldom consider the subjective expert weight due to the workload when the expert group is large.

**Figure 6:**
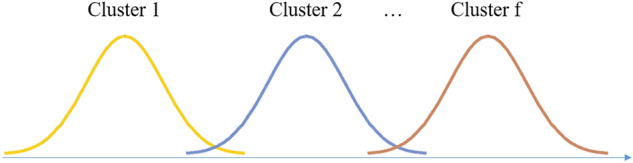
Distribution of experts in clusters.

In our work, we integrated the subjective and objective expert weights with a 2-layer cluster weight determination. The distribution of expert preferences in the clustering-based method is illustrated in Fig. 6. In the cluster layer (Layer 1), the Shannon entropy model can illustrate the organization of the condition of the clusters, but cannot well reflect the individual expert preferences. Hence, an aggregated method integrated by AHP and entropy weight model is proposed under this layer. In the subsection layer (Layer 2), the decision makers in the same cluster are the experts with similar knowledge and background, so their preferences should be highly consistent^40^. Under the circumstances, subjective pairwise comparison is omitted here, because on the one hand, it may largely increase workload, and on the other hand, the influence may not be obvious. Thus, an objective minimized variance model is used here.

The rest of this paper is organized as follows. In “Preliminaries”, relevant expert weight determination methods are reviewed. In “Methodology”, the conceptual framework of expert weight determination is presented. In “Case study”, a real-life example is implemented and related analysis is presented. In “Comparison of methods with and without using weight determination”, a comparison with and without using expert weight determination is discussed. In “Conclusions”, the conclusion is provided.

## Preliminaries

This study proposes a novel integrated expert weight determination method. Before presenting our method, some related expert weight determination methods are reviewed.

### The multiplicative AHP method

Multiplicative AHP is a significant subjective expert weight determination method. The method is easy to implement in real-life cases^23^. Similar to other subjective methods, the Multiplicative AHP determines the expert weight through pairwise comparisons. Initially, each expert in the decision maker group is allocated to assess every group member. It may lead to a personal upward bias^21,26^. To eliminate the error caused by the expert’s assessment, in Honert’s study, each expert’s comparisons with the expert him/herself are no longer counted. For example, each expert in the decision maker group with $G$ experts only needs to make $(G-1)$ comparisons.

The expert weight determination method of Multiplicative AHP in Honert’s approach can be summarized in the following steps^21^.

***Step 1***: Assume the expert group has $G$ members, DM $y$ is a member of the expert group. As is shown in Table 2, every expert is assigned to make a linguistic comparison between attributes by individual judgement using pre-provided words (Very strong preference / Strong preference / Definite preference / Weak preference / Indifferent). ${S}_{k}$ and ${S}_{j}$ represent the expert’s preference for the alternatives ${A}_{k}$ and ${A}_{j}$, respectively. Hence, comparison between ${S}_{k}$ and ${S}_{j}$ can be converted into a numerical value ${\delta }_{jky}$ by a geometric scale based on the content of Table 2. Then matrix ${\{r}_{jkd}\}$ can be obtained by the equation below:

1 $${r}_{jky}=\mathrm{exp}(\upgamma {\delta }_{jky})$$

where $\upgamma$ represents a scale parameter, with a frequently-used value of $\mathit{ln}2$. Next, approximate vector $p$ of stimulus values can be determined by the logarithmic least-squares method by Lootsma^47^. The vector $p$ minimizes

**Table 2:** Integer-valued index designating the gradations made by decision makers^24^.

Comparative judgement	Gradation index ${{\varvec{\delta}}}_{{\varvec{j}}{\varvec{k}}{\varvec{d}}}$
Very strong preference for ${S}_{k}$ versus ${S}_{j}$	− 8
Strong preference for ${S}_{k}$ versus ${S}_{j}$	− 6
Definite preference for ${S}_{k}$ versus ${S}_{j}$	− 4
Weak preference for ${S}_{k}$ versus ${S}_{j}$	− 2
Indifference between ${S}_{k}$ versus ${S}_{j}$	0
Weak preference for ${S}_{j}$ versus ${S}_{k}$	+ 2
Definite preference for ${S}_{j}$ versus ${S}_{k}$	+ 4
Strong preference for ${S}_{j}$ versus ${S}_{k}$	+ 6
Very strong preference ${S}_{j}$ versus ${S}_{k}$	+ 8

2 $$\sum_{j<k}\sum_{y\in {S}_{jk}}{\left(\mathrm{ln}{r}_{jky}-\mathrm{ln}{p}_{j}+\mathrm{ln}{p}_{k}\right)}^{2}$$

***Step 2***: Substituting ${w}_{j}=\mathrm{ln}{p}_{j}$,${w}_{k}=\mathrm{ln}{p}_{k}$ and ${q}_{jky}=\mathrm{ln}{r}_{jky}=\upgamma {\delta }_{jky}$, the function transforms to

3 $$\sum_{j<k}{\sum\limits_{d\in {S}_{jk}}\left({q}_{jky}-{w}_{j}+{w}_{k}\right)}^{2}$$

The associated set of normal equations can be transferred by ${\mathrm{w}}_{j}$ to

4 $${\mathrm{w}}_{j}\sum\limits_{k=1,k\ne j}^{G}{N}_{jk}-{\mathrm{w}}_{k}\sum\limits_{k=1,k\ne j}^{G}{N}_{jk}=\sum\limits_{k=1,k\ne j}^{G}\sum\limits_{d\in {S}_{jk}}{q}_{jky}$$

where $j=\mathrm{1,2},\dots ,G$. The variate $j$ has the same value range in the following equations in this part.${N}_{jk}$ denotes the cardinality of ${S}_{jk}$. According to Lootsma, ${N}_{jk}=G-2$. $(G-1)$ comparisons are made for each expert, the maximum pairwise comparison is $(G-1)(\mathrm{G}-2)/2$, and Eq. (4) can be rewritten as:

5 $${\mathrm{w}}_{j}\left(\mathrm{G}-1\right)\left(\mathrm{G}-2\right)-\sum\limits_{k=1,k\ne j}^{G}\left(G-2\right){\mathrm{w}}_{k}=\sum\limits_{k=1,k\ne j}^{G}\sum\limits_{d\in {S}_{jk}}{q}_{jky}$$

The equation can be simplified as:

6 $${\mathrm{w}}_{j}\mathrm{G}\left(\mathrm{G}-2\right)-\left(G-2\right)\sum\limits_{k=1,k\ne j}^{G}{\mathrm{w}}_{k}=\sum\limits_{k=1,k\ne j}^{G}\sum\limits_{y=1,y\ne j}^{G}{q}_{jky}$$

***Step 3***: For any alternative ${A}_{k}$ and ${A}_{j}$, from Table 2, we have ${q}_{jky}=-{q}_{kjy}$, ${S}_{jj}$ is empty and ${q}_{jjy}=0$.

Assume each expert made all the comparisons, then $\sum\limits_{k=1,k\ne j}^{G}{\mathrm{w}}_{k}=0$, the equation can be written in the following form:

7 $${\mathrm{w}}_{j}=[{G\left(G-2\right)]}^{-1}\sum\limits_{k=1,k\ne j}^{G}\sum\limits_{y=1,y\ne j}^{G}{q}_{jky}$$

Thus the expert weight of decision makers ${p}_{j}$ can be computed by Eq. (8).

8 $${p}_{j}=\mathrm{exp}\left({\mathrm{w}}_{j}\right)={\prod }_{k=1,k\ne j}^{G}{\prod }_{y=1,y\ne j}^{G}\mathrm{exp}{(\gamma [{\mathrm{G}\left(\mathrm{G}-2\right)]}^{-1})}^{{\delta }_{jky}}$$

After normalization with the equation below, the subjective expert weight ${w}_{i}^{s}$ can be determined.

9 $${w}_{i}^{s}=\frac{{p}_{i}}{\sum {p}_{i}}$$

### 2-tuple linguistic and the model of the two-layer weight determination method

#### Definitions

As described in “Introduction”, the two-layer weight determination method is a critical cluster-based method. The 2-tuple linguistic (2TL) provides the environment of the two-layer method, and this part introduces the rationale to select 2TL. Due to the uncertain environment, fuzzy set^48^, rough set^49^, grey decision^50^, and some other extended methods are applied in MADM problems^51^. 2TL is a model based on the linguistic fuzzy set proposed by Herrera and Martínez^52^. A symbolic translation value $\mathrm{\alpha \epsilon }\left[-0.5,\right.\left.0.5\right)$ is introduced to the linguistic fuzzy set to describe the flexibility of words. Reviews and extended models of 2TL can be found in Martínez^53,54^ and Malhotra^55^. Xu proposed non-linear aggregation operators in decision making with uncertain linguistic information^56,57^. Wang and Hao^58^ introduced a new 2TL model based on ‘symbolic proportion’ to preserve the integrity of information, as the proportional 2-tuples can well illustrate the uncertainty of the linguistic judgement. As linguistic term sets distribute uniformly and unsymmetrically, Herrera^59^ defined the term sets as unbalanced linguistic term sets to demonstrate the non-linear thinking of human beings, and proposed a method integrated by a representative algorithm and a computational approach. As an effective method in MADM, the 2TL model has produced crucial results in various areas such as quality assessment^60^, web system design^61^ and group decision making^62^. The 2TL model perfectly matches most subjective problems as the linguistic variables have their advantage in expressing ‘approximate information’. For example, we can use the 2TL model to describe ‘how young the person is’, using the linguistic words ‘very young, young or not young’.

In design concept evaluation, experts are assigned to evaluate plans on a large number of different attributes. Some of the attributes do not have an exact value, and thus the evaluation of the experts may be subjective and imprecise. Approaches such as the House of Quality method with multi-point scale measurement and intuitive fuzzy set are proposed to tackle the challenge. However, the 2TL is suitable for design concept evaluation problems for two reasons. First, design concept evaluation is complex multi-attribute decision making based on the aggregation of the decision makers’ judgements, which belongs to the MADM problem. Second, experts prefer to make judgements in natural language with some adverbs of degree such as ‘very’, ‘extremely’ etc.^55^ In design concept evaluation, raw data and information may be uncertain, imprecise and vague, which well matches the category of 2TL model problem-solving, and the evaluation data can be used as raw data in some MADM methods such as TOPSIS and VIKOR.

The notions, terminology definitions, and related equations of 2TL are presented below.

##### Definition 1

Let $\mathrm{S}=\{{s}_{i}|i=\mathrm{0,1},2,\dots ,t\}$ set as a linguistic term set (LTS), let $\beta \in \left[0,t\right]$ be a numeric result of the LTSs and $t+1$ be the LTS cardinality. We have two values $i=\mathrm{round}(\beta )$ and $\alpha =\beta -i$, where $\alpha$ is called a symbolic translation, $i\in \left[0,t\right]$ and $\alpha \in [-\mathrm{0.5,0.5})$. $\mathrm{round}(.)$ is the round operation in the definition.

##### Definition 2

^52^ Let $\upbeta \in \left[0,t\right]$ be the aggregation of LTSs from S, $\mathrm{S}=\{{s}_{i}|i=\mathrm{0,1},2,\dots ,t\}$. The 2-tuple gives the same information of $\beta$ and can be described as:

10 $$\Delta :\left[0,g\right]\to \overline{S }$$

11 $$\Delta :\left(\upbeta \right)=({s}_{round\left(\beta \right)},\beta -1)$$

where $\overline{S }=S\times [-\mathrm{0.5,0.5})$ in expression (10), $\Delta$ is a function to obtain the 2-tuple linguistic information. With the help of value 0 as the “symbolic translation” in 2-tuple linguistic term demonstration, ${s}_{i}$ transfers to a 2-tuple $({s}_{i}, 0)$. Herrera^52^ also gives a comparison rule of 2-tuples. Assume $({s}_{i}, {\mathrm{\alpha }}_{1})$ and $({s}_{j}, {\mathrm{\alpha }}_{2})$ are both 2-tuples, then

- If $$\mathrm{i}<\mathrm{j}\Rightarrow({s}_{i}, {\mathrm{\alpha }}_{1})< ({s}_{j}, {\mathrm{\alpha }}_{2})$$;
- If $$\mathrm{i}=\mathrm{j}$$, then
  - If $${\mathrm{\alpha }}_{1}={\mathrm{\alpha }}_{2}\Rightarrow \left({s}_{i}, {\mathrm{\alpha }}_{1}\right)=({s}_{j}, {\mathrm{\alpha }}_{2})$$;
  - If $${\mathrm{\alpha }}_{1}<{\mathrm{\alpha }}_{2}\Rightarrow ({s}_{i}, {\mathrm{\alpha }}_{1})< ({s}_{j}, {\mathrm{\alpha }}_{2})$$;
  - If $${\mathrm{\alpha }}_{1}>{\mathrm{\alpha }}_{2}\Rightarrow \left({s}_{i}, {\mathrm{\alpha }}_{1}\right)> ({s}_{j}, {\mathrm{\alpha }}_{2})$$.

##### Definition 3

^52^ Let $\mathrm{S}=\{{s}_{i}|i=\mathrm{0,1},2,\dots ,t\}$. ${\Delta }^{-1}$ is a function restoring the 2-tuple $({s}_{i}, {\mathrm{\alpha }}_{i})$ to its numerical value $\beta \in \left[0,t\right]\subset R$, where

12 $${\Delta }^{-1}: S\times \left[-\mathrm{0.5,0.5}\right)\to \left[0,t\right]$$

13 $${\Delta }^{-1}:\left({s}_{i}, \alpha \right)=i+\alpha =\beta $$

##### Definition 4

^52^ Let $\mathrm{S}=\{{s}_{i}|i=\mathrm{0,1},2,\dots ,t\}$. Then $\mathrm{t}$ 2-tuples is denoted by $({s}_{1}, {\mathrm{\alpha }}_{1})$ to $({s}_{t}, {\mathrm{\alpha }}_{t})$. The 2-tuple arithmetic mean (TAM) is given as:

14 $$\mathrm{TAM}\left(\left({s}_{1}, {\mathrm{\alpha }}_{1}\right),\dots ,\left({s}_{t}, {\mathrm{\alpha }}_{t}\right)\right)=\Delta \left(\frac{1}{t}\sum\limits_{k=1}^{t}{\Delta }^{-1}\left({s}_{k}, {\mathrm{\alpha }}_{k}\right)\right)=\Delta (\frac{1}{t}\sum\limits_{k=1}^{t}{\beta }_{k})$$

where $\mathrm{\alpha }\in [-\mathrm{0.5,0.5})$ in Eq. (14).

##### Definition 5

^63^ The deviation between 2-tuples $\left({s}_{i}, {\mathrm{\alpha }}_{i}\right)$ and $\left({s}_{j}, {\mathrm{\alpha }}_{j}\right)$ can be described as:

15 $$d\left(\left({s}_{i}, {\mathrm{\alpha }}_{i}\right),\left({s}_{j}, {\mathrm{\alpha }}_{j}\right)\right)={\Delta }^{-1}\left({s}_{i}, {\mathrm{\alpha }}_{i}\right)-{\Delta }^{-1}\left({s}_{j}, {\mathrm{\alpha }}_{j}\right)={\beta }_{i}-{\beta }_{j}$$

Moreover, we can easily get the following results from Eq. (15):

- $$d\left(\left({s}_{i}, {\mathrm{\alpha }}_{i}\right),\left({s}_{j}, {\mathrm{\alpha }}_{j}\right)\right)=-d\left(\left({s}_{j}, {\mathrm{\alpha }}_{j}\right),\left({s}_{i}, {\mathrm{\alpha }}_{i}\right)\right)$$;
- $$d\left(\left({s}_{i}, {\mathrm{\alpha }}_{i}\right),\left({s}_{j}, {\mathrm{\alpha }}_{j}\right)\right)=d\left(\left({s}_{i}, {\mathrm{\alpha }}_{i}\right),\left({s}_{x}, {\mathrm{\alpha }}_{x}\right)\right)+d\left(\left({s}_{x}, {\mathrm{\alpha }}_{x}\right),\left({s}_{j}, {\mathrm{\alpha }}_{j}\right)\right)$$.

##### Definition 6

^52^ Let $\mathrm{S}=\{{s}_{i}|i=\mathrm{0,1},2,\dots ,t\}$,$\mathrm{t}$ 2-tuples is denoted by $({s}_{1}, {\mathrm{\alpha }}_{1})$ to $({s}_{t}, {\mathrm{\alpha }}_{t})$, let $\upomega ={({\upomega }_{1},\dots ,{\upomega }_{t})}^{T}$ as the weight vectors of S. The 2-tuple weight average (TWA) operator is given as:

16 $$\mathrm{TWA}\left(\left({s}_{1}, {\mathrm{\alpha }}_{1}\right),\dots ,\left({s}_{t}, {\mathrm{\alpha }}_{t}\right)\right)=\Delta \left(\sum\limits_{k=1}^{t}{{\upomega }_{k}\Delta }^{-1}\left({s}_{k}, {\mathrm{\alpha }}_{k}\right)\right)=\Delta (\sum\limits_{k=1}^{t}{{\upomega }_{k}\upbeta }_{k})$$

where $\mathrm{\alpha }\in [-\mathrm{0.5,0.5})$ in Eq. (16).

##### Definition 7

^40^ Let $\mathrm{S}=\{{s}_{i}|i=\mathrm{0,1},2,\dots ,t\}$. A 2-tuple matrix is expressed as $\mathrm{B}={({b}_{ij})}_{m\times n}$, where ${b}_{ij}=\left({s}_{ij},{\alpha }_{ij}\right), {s}_{ij}\in S$,$\mathrm{\alpha }\in [-\mathrm{0.5,0.5})$. Let $\mathrm{Cov}(\mathrm{a},\mathrm{b})$ be the covariance between a and b, let 2-tuple $\overline{{b }_{j}}=\left(\overline{{s }_{j}},\overline{{\alpha  }_{j}}\right)$, let $\mathrm{d}[\left({s}_{i}, {\mathrm{\alpha }}_{1}\right),\left({s}_{j}, {\mathrm{\alpha }}_{2}\right)]$ be the deviation between $\left({s}_{i}, {\mathrm{\alpha }}_{1}\right)$ and $\left({s}_{j}, {\mathrm{\alpha }}_{2}\right)$. Then

17 $$\mathrm{Cov}\left({b}_{j},{b}_{k}\right)=\frac{1}{m}\sum\limits_{i=1}^{m}\left[d\left({b}_{ij},\overline{{b }_{j}}\right)\times d\left({b}_{ik},\overline{{b }_{k}}\right)\right]=\frac{1}{m}\sum\limits_{i=1}^{m}\left[d\left({s}_{ij},{\alpha }_{ij}\right),\left(\overline{{s }_{j}},\overline{{\alpha  }_{j}}\right)\times d\left({b}_{ik},\overline{{b }_{k}}\right)\right]=\frac{1}{m}\sum\limits_{i=1}^{m}\left[\left({\upbeta }_{ij}-\frac{1}{m}\sum\limits_{i=1}^{m}{\upbeta }_{ij}\right)\times \left({\upbeta }_{ik}-\frac{1}{m}\sum\limits_{i=1}^{m}{\upbeta }_{ik}\right)\right]$$

When $\mathrm{j}=\mathrm{k}$, let ${\sigma }_{j}^{2}$ be the variance deviation of ${b}_{j}$, then have ${\sigma }_{j}^{2}=\mathrm{Cov}\left({b}_{j},{b}_{j}\right)$. The equation above can be converted to:

18 $${\sigma }_{j}^{2}=\frac{1}{m}\sum\limits_{i=1}^{m}{\left({\upbeta }_{ij}-\frac{1}{m}\sum\limits_{i=1}^{m}{\upbeta }_{ij}\right)}^{2}$$

where $$\mathrm{j}=\mathrm{1,2},\dots ,\mathrm{n}$$.

#### Computing process

The computing process of the two-layer determination method is described as:

Assume $S=\{{s}_{i}|i=\mathrm{0,1},2,\dots ,u\}$ is an LTS. The criteria (attribute) group and the expert group are expressed as $C=\{{c}_{1},{c}_{2},\dots ,{c}_{n}\}$ and $E=\{{e}_{1},{e}_{2},\dots ,{e}_{m}\}$ respectively. $A=\{{A}_{1},\dots ,{A}_{l}\}$ represented as *l* alternatives. The experts (*E*) are assigned to evaluate the alternatives (*A*) according to the criteria (*C*) using the LTS (*S*). The experts are required to give their evaluation using linguistic terms. As for expert ${e}_{k}$, the decision matrix ${X}_{k}$ is described as:

19 $${X}_{k}={({x}_{ij}^{k})}_{l\times n}$$

where $i,j$ and $k$ represent the index of alterative (${\mathrm{A}}_{i}$), criteria (${\mathrm{c}}_{j}$) and expert (${\mathrm{e}}_{k}$) respectively. In the two-layer method, *m* experts can be divided into *f* clusters, expressed as $G=\{{G}_{1},{G}_{2},\dots ,{G}_{f}\}$. There are ${m}_{y}$ experts in the cluster ${G}_{y}$, where $\sum\limits_{y=1}^{f}{m}_{y}=m$ and $y\le f$.

***Step*** ***1:*** Minimized variance model in layer 2.

The minimized variance model relies on minimizing the total variance preference in a cluster. The matrix ${X}_{k}$ can be transferred into 2-tuple ${B}_{k}$ as follows:

20 $${B}_{k}={({b}_{ij}^{k})}_{l\times n}$$

where ${b}_{ij}^{k}$ is a 2-tuple.

In the second layer, assume expert ${e}_{k}$ is the ${p}^{th}$ expert in the cluster ${G}_{y}$, we denote by ${B}_{yp}$ the ${p}^{th}$ decision matrix in ${G}_{y}$. Hence, we can rewrite Eq. (20) as:

21 $${B}_{yp}={({b}_{ij}^{yp})}_{l\times n}$$

where $p\le {m}_{y}$. To make the computing process simple, the matrix of an expert is converted to a vector:

22 $${F}_{yp}=\left({b}_{11}^{yp},\dots ,{b}_{1n}^{yp},\dots ,{b}_{l1}^{yp},\dots ,{b}_{ln}^{yp}\right)=({F}_{1}^{yp},\dots ,{F}_{t}^{yp})$$

where $t=l\times n$. The decision matrix of cluster ${G}_{y}$ is

23 $${F}^{y}={({F}_{ij}^{y})}_{{m}_{y}l}=\left[\begin{array}{ccc}{F}_{1}^{y1}& \dots & {F}_{t}^{y1}\\ \vdots & \ddots & \vdots \\ {F}_{1}^{y{m}_{y}}& \dots & {F}_{t}^{y{m}_{y}}\end{array}\right]$$

To get the optimized solution, it is required to make the sum of the variance of the attributes weight small.

According to Definition 7, we can get the weight evaluation of cluster ${G}_{y}$ using Eqs. (24) to (27):

24 $$ \begin{aligned}{\sigma }_{yj}^{2}&=\frac{1}{{m}_{y}}\sum\limits_{i=1}^{{m}_{y}}{\left[d\left({\lambda }_{yi}*{F}_{ij}^{y},{\overline{F} }_{j}^{y}\right)\right]}^{2}\\ &=\frac{1}{{m}_{y}}\sum\limits_{i=1}^{{m}_{y}}{[d({\lambda }_{yi}*{F}_{ij}^{y},\frac{1}{{m}_{y}}TWA(\left({s}_{1j}^{y},0\right),\left({s}_{2j}^{y},0\right),\dots ,({s}_{{m}_{y}j}^{y},0))]}^{2}\\ &=\frac{1}{{m}_{y}}\sum\limits_{i=1}^{{m}_{y}}{\left[{\Delta }^{-1}\left(\Delta \left({\lambda }_{yi}{\Delta }^{-1}\left({s}_{ij}^{y},0\right)\right)\right)-{\Delta }^{-1}\left(\Delta \left(\frac{1}{{m}_{y}}\left(\sum\limits_{i=1}^{{m}_{y}}{\lambda }_{yi}{\Delta }^{-1}\left({s}_{ij}^{y},0\right)\right)\right)\right)\right]}^{2}\\ &=\frac{1}{{m}_{y}}\sum\limits_{i=1}^{{m}_{y}}{\left[{\lambda }_{yi}{\beta }_{ij}-\frac{1}{{m}_{y}}\left(\sum\limits_{i=1}^{{m}_{y}}{\lambda }_{yi}{\beta }_{ij}\right)\right]}^{2}\end{aligned} $$

where ${\overline{F} }_{j}^{y}$ is the arithmetic mean of ${F}_{ij}^{y}$, and ${\lambda }_{yi}={({\lambda }_{1},{\lambda }_{2},\dots ,{\lambda }_{{m}_{y}})}^{T}$ indicates the weight vectors of experts in cluster ${G}_{y}$, and $\sum\limits_{i=1}^{{m}_{y}}{\lambda }_{yi}=1$. The definition of symbol * in this equation is defined as:

25 $${\lambda }_{yi}*{F}_{ij}^{y}=\Delta \left({\lambda }_{yi}{\Delta }^{-1}\left({s}_{ij}^{y},0\right)\right)$$

The summary of variances is:

26 $$sum\left({h}_{y}\left({\lambda }_{y}\right)\right)=\sum\limits_{j=1}^{t}{\sigma }_{yj}^{2}=\sum\limits_{j=1}^{t}\frac{1}{{m}_{y}}\sum\limits_{i=1}^{{m}_{y}}{\left[{\lambda }_{yi}{\beta }_{ij}-\frac{1}{{m}_{y}}\left(\sum\limits_{i=1}^{{m}_{y}}{\lambda }_{yi}{\beta }_{ij}\right)\right]}^{2}$$

The optimization model is shown below:

27 $$\begin{array}{c}\mathrm{min}{h}_{y}\left({\lambda }_{y}\right)=\sum\limits_{j=1}^{t}{\sigma }_{yj}^{2} \\ s.t.\left\{\begin{array}{c}\sum\limits_{i=1}^{{m}_{y}}{\lambda }_{yi}=1 \\ {\lambda }_{yi}\ge 0,i=\mathrm{1,2},\dots ,x\end{array}\right.\end{array}$$

Then, the optimal weight value of variate ${\lambda }_{y}$ can be determined as ${\lambda }_{y}^{*}=({\lambda }_{1},{\lambda }_{2},\dots ,{\lambda }_{{m}_{y}})$.

***Step*** ***2:*** Entropy weight model in Layer 1.

In the first layer, the TAM operator is applied in the entropy weight model. As the basic equation of entropy, we have:

28 $${Z}_{y}=-k\sum\limits_{j=1}^{n}{p}_{ij}\mathrm{ln}{p}_{ij}$$

where the constant *k* is calculated as $k=1/\mathrm{ln}n$. Let $D={({d}_{yj})}_{f\times n}$ as the decision matrix of the clusters. The equation above can be converted to:

29 $${Z}_{y}=-\frac{1}{\mathrm{ln}n}\sum\limits_{j=1}^{n}(\frac{{\Delta }^{-1}{d}_{yj}}{\sum\limits_{j=1}^{n}{\Delta }^{-1}{d}_{yj}}\mathrm{ln}\frac{{\Delta }^{-1}{d}_{yj}}{\sum\limits_{j=1}^{n}{\Delta }^{-1}{d}_{yj}})$$

Let ${w}_{y}={({w}_{1},{w}_{2},\dots ,{w}_{f})}^{T}$ be the weight vector of clusters. We can compute the cluster weight by the equation below:

30 $${w}_{y}=\frac{1-{Z}_{y}}{f-\sum\limits_{y=1}^{f}{Z}_{y}}$$

***Step*** ***3:*** Expert weight calculation.

By now, the expert weight in cluster ${\lambda }_{yi}^{*}$ and the cluster weight ${w}_{y}$ are determined, and the *i*^*th*^ expert weight in cluster *G*_*y*_, the expert weight *w*_*e*_ can be obtained by multiplying the two weights.

31 $${w}_{e}={\lambda }_{yi}^{*}\times {w}_{y}$$

Thus, the expert weight can be determined by the two-layer weight determination method.

## Methodology

**Figure 7:**
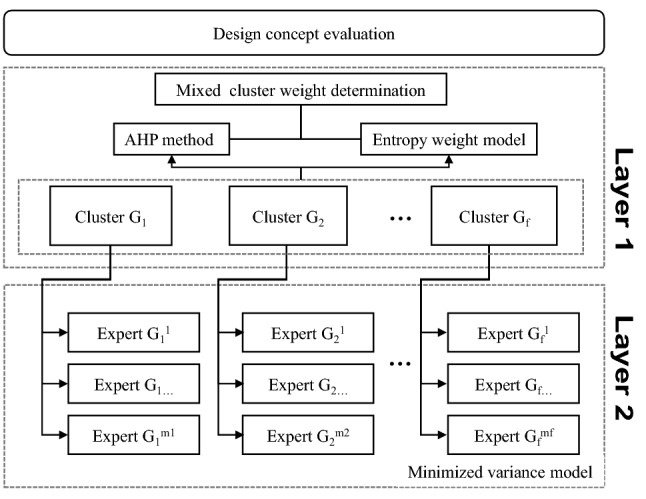
The framework of the proposed method.

In design concept evaluation, attributes are selected from multiple dimensions, such as shape, color, ergonomics, material and manufacturing technology, etc. The experts are categorized into clusters depending on their different backgrounds. Therefore, a two-layer expert weight determination is a good solution to design concept evaluation in NPD. However, there are still some drawbacks to this method. The complexity of different products varies, experts in the customer cluster may not be familiar with how the product works, and they may make judgements relying only on their experiences. Moreover, if the experts’ weights merely depend on the objective method, the subjective preferences of the experts are ignored. Thus, an integrated two-layer expert weight determination approach is proposed, as shown in Fig. 7. In the first layer, we used a mixed weight determination method based on the AHP and entropy weight model. In the second layer, a minimized variance model as mentioned above was applied. After that, the final expert weight is determined by combining the two layers’ results. The proposed method is illustrated below.

***Step*** ***1:*** In this step, a 9 LTS *S* is constructed according to the 2TL environment.

$${S}^{9}=\left\{{s}_{0}, { s}_{1}, { s}_{2},{ s}_{3},{ s}_{4},{ s}_{5},{ s}_{6},{ s}_{7},{ s}_{8}\right\}=\{none,extremely poor,very poor,poor, neutral,good,very good, extremely good,perfect\}$$

Then, the experts are assigned to give their preferences by the languages given in the LTS. The criteria (attribute) group and the expert group are expressed as $C=\{{c}_{1},{c}_{2},\dots ,{c}_{n}\}$ and $E=\{{e}_{1},{e}_{2},\dots ,{e}_{m}\}$ respectively. $A=\{{A}_{1},\dots ,{A}_{l}\}$ represents *l* alternatives. Then, *m* experts can be divided into *f* clusters, expressed as $G=\{{G}_{1},{G}_{2},\dots ,{G}_{f}\}$. There are ${m}_{y}$ experts in the cluster ${G}_{y}$, where $\sum\limits_{y=1}^{f}{m}_{y}=m$ and $y\le f$.

The experts are assigned to give their evaluation using linguistic terms. As for expert ${e}_{k}$, the decision matrix ${A}_{k}$ is described as:

32 $${A}_{k}={({a}_{ij}^{k})}_{l\times n}$$

where $i,j$ and $k$ represent the index of alternative (${A}_{i}$), criteria (${\mathrm{c}}_{j}$) and expert (${\mathrm{e}}_{k}$), respectively. The matrix ${A}_{k}$ can also be shown with 2-tuple:

33 $${B}_{k}={({b}_{ij}^{k})}_{l\times n}$$

where ${b}_{ij}^{k}$ is a 2-tuple. In the second layer, assume expert ${e}_{k}$ is the ${p}^{th}$ expert in the cluster ${G}_{y}$, we denote by ${B}_{yp}$ the ${p}^{th}$ decision matrix in ${G}_{y}$. Hence, we can rewrite Eq. (33) as:

34 $${B}_{yp}={({b}_{ij}^{yp})}_{l\times n}$$

where $p\le {m}_{y}$. To make the computing process simple, the matrix of an expert is converted to a vector:

35 $${F}_{yp}=\left({b}_{11}^{yp},\dots ,{b}_{1n}^{yp},\dots ,{b}_{m1}^{yp},\dots ,{b}_{mn}^{yp},\right)=({F}_{1}^{yp},\dots ,{F}_{t}^{yp})$$

where $t=l\times n$. The decision matrix of the designer cluster is:

36 $${F}^{y}={({F}_{ij}^{y})}_{{m}_{y}l}=\left[\begin{array}{ccc}{F}_{1}^{y1}& \dots & {F}_{t}^{y1}\\ \vdots & \ddots & \vdots \\ {F}_{1}^{y{m}_{y}}& \dots & {F}_{t}^{y{m}_{y}}\end{array}\right]$$

Thus, the decision matrix of layer 2 can be represented as $F={({F}^{1}, {F}^{2},\dots , {F}^{f})}^{T}$.

***Step*** ***2:*** The weight of experts in the corresponding cluster is calculated by the minimized variate model in the second layer.

Generally, experts in the same cluster have a similar background, which means the experts have a high possibility of having similar preferences for the alternatives. It is required to minimize the variance of the attributes of the clusters. To get the optimized solution, it is required to make the sum of the variance of the attributes weight small. Using Eqs. (37) to (40), we can get the weight evaluation of the designer cluster:

37 $$ \begin{aligned}{\sigma }_{yj}^{2}&=\frac{1}{x}\sum\limits_{i=1}^{x}{\left[d\left({\lambda }_{yi}*{F}_{ij}^{y},{\overline{F} }_{j}^{y}\right)\right]}^{2}=\frac{1}{x}\sum\limits_{i=1}^{x}{[d({\lambda }_{yi}*{F}_{ij}^{y},\frac{1}{r}TWA(\left({s}_{1j}^{y},0\right),\left({s}_{2j}^{y},0\right),\dots ,({s}_{rj}^{y},0))]}^{2}\\&=\frac{1}{x}\sum\limits_{i=1}^{x}{\left[{\Delta }^{-1}\left(\Delta \left({\lambda }_{yi}{\Delta }^{-1}\left({s}_{ij}^{y},0\right)\right)\right)-{\Delta }^{-1}\left(\Delta \left(\frac{1}{x}\left(\sum\limits_{i=1}^{x}{\lambda }_{yi}{\Delta }^{-1}\left({s}_{ij}^{y},0\right)\right)\right)\right)\right]}^{2}\\ &=\frac{1}{x}\sum\limits_{i=1}^{x}{\left[{\lambda }_{yi}{\beta }_{ij}-\frac{1}{x}\left(\sum\limits_{i=1}^{x}{\lambda }_{yi}{\beta }_{ij}\right)\right]}^{2}\end{aligned} $$

where ${\overline{F} }_{j}^{y}$ is the arithmetic mean of ${F}_{ij}^{y}$, and ${\lambda }_{yi}={({\lambda }_{1},{\lambda }_{2},\dots ,{\lambda }_{x})}^{T}$ indicates the weight vectors of experts in cluster ${G}_{y}$, and the summary of weight vectors is 1. The definition of symbol * in this equation is:

38 $${\lambda }_{yi}*{F}_{ij}^{y}=\Delta \left({\lambda }_{yi}{\Delta }^{-1}\left({s}_{ij}^{y},0\right)\right)$$

The summary of variances is:

39 $$sum\left({h}_{y}\left({\lambda }_{y}\right)\right)=\sum\limits_{j=1}^{t}{\sigma }_{yj}^{2}=\sum\limits_{j=1}^{t}\frac{1}{x}\sum\limits_{i=1}^{x}{\left[{\lambda }_{yi}{\beta }_{ij}-\frac{1}{x}\left(\sum\limits_{i=1}^{x}{\lambda }_{yi}{\beta }_{ij}\right)\right]}^{2}$$

The optimization model is shown below:

40 $$\begin{array}{c}\mathrm{min}{h}_{y}\left({\lambda }_{y}\right)=\sum\limits_{j=1}^{t}{\sigma }_{yj}^{2} \\ s.t.\left\{\begin{array}{c}\sum\limits_{i=1}^{x}{\lambda }_{yi}=1 \\ {\lambda }_{yi}\ge 0,i=\mathrm{1,2},\dots ,x\end{array}\right.\end{array}$$

Then, the optimal weight value of variate ${\lambda }_{y}$ can be determined as ${\lambda }_{y}^{*}=({\lambda }_{1},{\lambda }_{2},\dots ,{\lambda }_{x})$. Similarly, the optimal weight value of the rest of the clusters in the second layer can be determined in the same way.

***Step*** ***3:*** Next, we determined the cluster weight in the first layer by a hybrid method. In this step, we use the entropy weight model to determine the objective weight of clusters.

In the first layer, the TAM operator is applied in the entropy weight model. For the cluster ${G}_{y}$, as the basic equation of entropy, we have:

41 $${Z}_{y}=-k\sum\limits_{j=1}^{n}{p}_{ij}\mathrm{ln}{p}_{ij}$$

where the constant *k* is calculated as $k=1/\mathrm{ln}n$*.* Let $D={({d}_{Dj})}_{f\times n}$ as the decision matrix of clusters. The equation above can be converted to:

42 $${Z}_{y}=-\frac{1}{\mathrm{ln}n}\sum\limits_{j=1}^{n}\left(\frac{{\Delta }^{-1}{d}_{yj}}{\sum\limits_{j=1}^{n}{\Delta }^{-1}{d}_{yj}}\mathrm{ln}\frac{{\Delta }^{-1}{d}_{yj}}{\sum\limits_{j=1}^{n}{\Delta }^{-1}{d}_{yj}}\right)$$

Let ${\mathrm{w}}^{o}={({w}_{1}^{o},{w}_{2}^{o},\dots ,{w}_{f}^{o})}^{T}$ be the weight vector of clusters. Then

43 $${w}_{y}^{o}=\frac{1-{Z}_{y}}{f-\sum\limits_{y=1}^{f}{Z}_{y}}$$

The result of ${\mathrm{w}}^{o}$ can be computed as the weight vector of clusters by the objective method.

***Step*** ***4:*** Here in this step, a subjective method is also implemented in cluster weight determination. The experts are assigned to assess the importance of three groups by the AHP method. They give their comparison of preferences among the clusters.

First, the experts are assigned to compare the clusters, the comparison among ${G}_{1},{G}_{2},\dots ,{G}_{f}$. Every expert is assigned to make a linguistic comparison between clusters by individual judgement using specified words (Very strong importance / Strong importance / Definite importance / Weak importance / Indifferent). The comparisons made by experts in ${G}_{y}$ between cluster ${G}_{\alpha }$ and ${G}_{\beta }$ are converted into a gradation index by geometric scale shown in Table 3. The comparison can be recorded in the comparative table in the form of Table 3. Where ${G}_{y}^{x}({G}_{\alpha }/{G}_{\beta })$ represents the comparison of ${G}_{\alpha }/{G}_{\beta }$ made by the ${x}^{th}$ expert in cluster ${G}_{y}$. After that, the arithmetic means of the comparison by cluster can be computed, which is also shown in Table 4. ${\delta }_{\alpha \beta y}$ denotes the arithmetic mean of the comparison ${G}_{\alpha }/{G}_{\beta }$ made by experts in cluster ${G}_{y}$, then we have ${\delta }_{\alpha \beta y}={G}_{y}({G}_{\alpha }/{G}_{\beta })$.

**Table 3:** Integer-valued cluster important judgement designating the gradations made by decision makers.

Comparative judgement	Gradation index
Very strong importance for ${G}_{\alpha }$ versus ${G}_{\beta }$	− 8
Strong importance for ${G}_{\alpha }$ versus ${G}_{\beta }$	− 6
Definite importance for ${G}_{\alpha }$ versus ${G}_{\beta }$	− 4
Weak importance for ${G}_{\alpha }$ versus ${G}_{\beta }$	− 2
Indifference between ${G}_{\alpha }$ versus ${G}_{\beta }$	0
Weak importance for ${G}_{\beta }$ versus ${G}_{\alpha }$	+ 2
Definite importance for ${G}_{\beta }$ versus ${G}_{\alpha }$	+ 4
Strong importance for ${G}_{\beta }$ versus ${G}_{\alpha }$	+ 6
Very strong importance ${G}_{\beta }$ versus ${G}_{\alpha }$	+ 8

**Table 4:** Form of the data recorded in the table based on pairwise comparative judgements.

Cluster ${G}_{1}$				Cluster ${G}_{2}$		$\dots$	Cluster ${G}_{f}$			
	${{\varvec{G}}}_{1}$**/**${{\varvec{G}}}_{2}$	$\dots$	${{\varvec{G}}}_{{\varvec{f}}-1}$**/**${{\varvec{G}}}_{{\varvec{f}}}$		$\dots$	$\dots$		${{\varvec{G}}}_{1}$**/**${{\varvec{G}}}_{2}$	$\dots$	${{\varvec{G}}}_{{\varvec{f}}-1}$**/**${{\varvec{G}}}_{{\varvec{f}}}$
1	${G}_{1}^{1}({G}_{1}/{G}_{2})$	$\dots$	${G}_{1}^{1}({G}_{f-1}/{G}_{f})$	**1**	$\dots$	$\dots$	1	${G}_{f}^{1}({G}_{1}/{G}_{2})$	$\dots$	${G}_{f}^{1}({G}_{f-1}/{G}_{f})$
2	${G}_{1}^{2}({G}_{1}/{G}_{2})$		${G}_{1}^{2}({G}_{f-1}/{G}_{f})$	**2**			2	${G}_{f}^{2}({G}_{1}/{G}_{2})$		${G}_{f}^{2}({G}_{f-1}/{G}_{f})$
**…**	…		…	**…**			**…**	…		…
m_1_	${G}_{1}^{m1}({G}_{1}/{G}_{2})$		${G}_{1}^{m1}({G}_{f-1}/{G}_{f})$	***m***_**2**_			m_f_	${G}_{f}^{mf}({G}_{1}/{G}_{2})$		${G}_{f}^{mf}({G}_{f-1}/{G}_{f})$
AVE	${G}_{1}({G}_{1}/{G}_{2})$	$\dots$	${G}_{1}({G}_{f-1}/{G}_{f})$	**AVE**	$\dots$	$\dots$	AVE	${G}_{f}({G}_{1}/{G}_{2})$	$\dots$	${G}_{f}({G}_{f-1}/{G}_{f})$

The comparison matrix of clusters ${\{r}_{\alpha \beta y}\}$ can be determined by the equation below:

44 $${r}_{\alpha \beta y}=\mathrm{exp}(\upgamma {\delta }_{\alpha \beta y})$$

where $\upgamma$ represents a scale parameter, with a frequently-used value of $\mathrm{ln}2$. Next, the approximate vector $p$ of stimulus values can be determined by the logarithmic least-squares method by Lootsma^47^. The vector $p$ minimizes

45 $$\sum_{\alpha <\beta }\sum_{{G}_{y}\in G}{\left(\mathrm{ln}{r}_{\alpha \beta y}-\mathrm{ln}{p}_{\alpha }+\mathrm{ln}{p}_{\beta }\right)}^{2}$$

where ${p}_{\alpha }$ and ${p}_{\beta }$ represent the relative power of ${G}_{\alpha }$ and ${G}_{\beta }$ made by ${G}_{y}$, respectively. Assume ${q}_{\alpha \beta y}=\mathrm{ln}{r}_{\alpha \beta y}$ and ${w}_{\alpha }=\mathrm{ln}{p}_{\alpha }$, expression (45) can be described as:

46 $$\sum_{\alpha <\beta }{\sum\limits_{{G}_{y}\in G}\left({q}_{\alpha \beta y}-{w}_{\alpha }+{w}_{\beta }\right)}^{2}$$

The associated set of normal equations can be transferred by ${w}_{\alpha }$ to:

47 $${w}_{\alpha }\sum\limits_{\alpha =1,\alpha \ne \beta }^{f}{N}_{\alpha \beta }-{\mathrm{w}}_{\beta }\sum\limits_{\beta =1,\beta \ne \alpha }^{f}{N}_{\alpha \beta }=\sum\limits_{\beta =1,\beta \ne \alpha }^{f}\sum\limits_{{G}_{y}\in G}{q}_{\alpha \beta y}$$

where $\alpha =\mathrm{1,2},\dots ,f$, and the variate $\alpha$ has the same value range in the following equations in this part.${N}_{\alpha \beta }$ denotes the cardinality of $G$. According to Lootsma, we have ${N}_{\alpha \beta }=f-2$. $(f-1)$ comparison made for each expert, the maximum pairwise comparisons are $(f-1)(f-2)/2$, and Eq. (47) can be rewritten as:

48 $${\mathrm{w}}_{j}\left(f-1\right)\left(f-2\right)-\sum\limits_{\beta =1,\beta \ne \alpha }^{f}\left(f-2\right){\mathrm{w}}_{k}=\sum\limits_{\beta =1,\beta \ne \alpha }^{f}\sum\limits_{{G}_{y}\in G}{q}_{\alpha \beta y}$$

The equation can be simplified as:

49 $${\mathrm{w}}_{j}f\left(f-2\right)-\left(f-2\right)\sum\limits_{\beta =1,\beta \ne \alpha }^{f}{\mathrm{w}}_{k}=\sum\limits_{\beta =1,\beta \ne \alpha }^{f}\sum\limits_{y=1,y\ne \alpha }^{f}{q}_{\alpha \beta y}$$

For any cluster ${G}_{\alpha }$ and ${G}_{\beta }$, from Table 2, we have ${q}_{\alpha \beta y}=-{q}_{\beta \alpha y}$, ${G}_{\alpha \alpha }$ is empty and ${q}_{\alpha \alpha y}=0$.

Assume each expert made all the comparisons, then $\sum\limits_{\beta =1,\beta \ne \alpha }^{f}{\mathrm{w}}_{k}=0$, the equation can be written in the following form:

50 $${w}_{j}=[{f\left(f-2\right)]}^{-1}\sum\limits_{\beta =1,\beta \ne \alpha }^{f}\sum\limits_{y=1,y\ne \alpha }^{f}{q}_{\alpha \beta y}$$

Thus the expert weight of decision makers ${p}_{\alpha }$ can be computed by Eq. (48).

51 $${p}_{\alpha }=\mathit{exp}\left({w}_{j}\right)={\prod }_{\beta =1,\beta \ne \alpha }^{f}{\prod }_{y=1,y\ne \alpha }^{f}exp{(\gamma [{f\left(f-2\right)]}^{-1})}^{{\delta }_{\alpha \beta y}}$$

After normalization with the equation below, the subjective expert weight ${w}_{i}^{s}$ can be determined.

52 $${w}_{i}^{s}=\frac{{p}_{i}}{\sum {p}_{i}}$$

***Step*** ***5:*** In Step 3 and Step 4 above, the objective cluster weight and the subjective cluster weights were determined. Here in this step, combined weights are determined using the equation below to get the combined weight of clusters.

53 $${\mathrm{w}}_{ci}=\frac{{({w}_{i}^{s})}^{\mu }{({w}_{i}^{o})}^{(1-\mu )}}{\sum\limits_{1}^{3}{({w}_{i}^{s})}^{\mu }{({w}_{i}^{o})}^{(1-\mu )}}$$

where $\mu$ is the adjusting coefficient, here $\mu \in [\mathrm{0,1}]$ represents the superiority of the subjective method over the objective method in the combination. When $\mu >0.5$, the subjective determination of the DM group is superior, on the contrary, when $\mu <0.5$, the objective method is superior, and when $\mu =0.5$, both methods made the same contribution in the cluster weight determination.

***Step*** ***6:*** Thus, the final weight of each expert ${\upomega }_{ui}$ can be computed by:

54 $${\upomega }_{ui}={\mathrm{w}}_{ci}{\lambda }_{u}^{*}$$

### Ethical approval

This article does not contain any studies with human participants or animals performed by any of the authors.

### Informed consent

No informed consent was required, because the data are anonymized.

## Case study

In our study, the new two-layer expert weight determination method is applied in optimization of the cabin design plan for a mid-sized cruise ship. Before the evaluation, three design schemes are proposed, and a decision needs to be made from the three alternatives. To make the decision, a 10 attribute criteria index shown in Table 5 is fixed to determine the alternatives.

**Table 5:** Attributes defined in the case study.

Attribute	Specification	Attribute	Specification
C_1_	Planning Compliance	C_6_	Style and trend
C_2_	User acceptance	C_7_	Reasonable placement of furniture
C_3_	Design humanized	C_8_	Innovation and competitiveness
C_4_	Design aesthetics	C_9_	Cost matches quality control
C_5_	Ergonomics	C_10_	Development feasibility

A 30-expert group is formed with 10 cruise ship interior designers, 10 cruise ship manufacturing specialists and 10 customers with over two cruise trip experiences. The raw data is shown in Appendix 1.

First, according to Eqs. (32) to (40), a minimized variance mode is established. Then the optimized lambdas(λ) are determined by the software Lingo. The corresponding weight of experts in each group are shown in Table 6.

**Table 6:** Weight of experts in the designer, manufacturer and customer clusters.

λ	Designer	Manufacturer	Customer
λ1	0.0994	0.0941	0.0884
λ2	0.1001	0.1042	0.0851
λ3	0.1002	0.1020	0.0904
λ4	0.1044	0.0992	0.0838
λ5	0.0982	0.1040	0.1073
λ6	0.1012	0.0984	0.1186
λ7	0.1060	0.0960	0.1107
λ8	0.0991	0.0982	0.1037
λ9	0.0945	0.1051	0.1048
λ10	0.0967	0.0987	0.1073

After that, according to the TAM operator from **Definition 3**, the decision matrix *D* is shown in Table 7.

**Table 7:** Decision matrix *D.*

	Alternative 1	Alternative 2	Alternative 3
Designers	(S_6_, − 0.29)	(S_7_, − 0.19)	(S_7_, − 0.17)
Manufacturers	(S_6_, − 0.21)	(S_6_, + 0.42)	(S_6_, + 0.42)
Customers	(S_6_, + 0.14)	(S_7_, − 0.43)	(S_7_, − 0.43)

We can calculate the objective weight of the clusters ${\mathrm{w}}^{o}=(\mathrm{0.424,0.373,0.203})$.

We get the pairwise judgement about the importance of the clusters from Appendix 2. The data in Table 8 is the arithmetic mean calculated from subjective pairwise judgements using the weighting method in each cluster. Here in the table, we leave the cell blank when the cluster is compared against itself. For other cells, the symbol “-” means that the corresponding row or column is not permitted to be compared with others. The normal equation can be expressed as:

**Table 8:** Hypothetical subjective pairwise judgements of clusters.

	DC			MC			CC		
DC				–	–	–0.4	–	–1	–
MC	–	–	0.4				–1.8	–	–
CC	–	1	–	1.8	–	–			

55 $$3{\mathrm{w}}_{\alpha }-\sum\limits_{\beta =1,\beta \ne \alpha }^{3}{\mathrm{w}}_{\beta }=\sum\limits_{\beta =1,\beta \ne \alpha }^{3}\sum\limits_{y=1,y\ne \alpha }^{3}{q}_{\alpha \beta y},\alpha =\mathrm{1,2},3$$

In the equation, ${\mathrm{q}}_{\alpha \beta y}=\upgamma {\updelta }_{\alpha \beta y}$, Eq. (55) can be simplified as:

For DC, $\alpha =1$, we have

56 $$2{\mathrm{w}}_{1}-{\mathrm{w}}_{2}-{\mathrm{w}}_{3}=\upgamma \sum\limits_{\beta =1,\beta \ne 1}^{3}\sum\limits_{y=1,y\ne 1}^{3}{\updelta }_{1\beta y}=(0.4+1)\upgamma $$

For MC, $\alpha =2$, we have

57 $$-{\mathrm{w}}_{1}+2{\mathrm{w}}_{2}-{\mathrm{w}}_{3}=\upgamma \sum\limits_{\beta =1,\beta \ne 2}^{3}\sum\limits_{y=1,y\ne 2}^{3}{\updelta }_{2\beta y}=(-0.4+1.8)\upgamma $$

For CC, $\alpha =3$, we have

58 $$-{\mathrm{w}}_{1}-{\mathrm{w}}_{2}+2{\mathrm{w}}_{3}=\upgamma \sum\limits_{\beta =1,\beta \ne 3}^{3}\sum\limits_{y=1,y\ne 3}^{3}{\updelta }_{3\beta y}=(-1\pm 1.8)\upgamma $$

Thus, we can compute ${\mathrm{w}}_{\alpha }$ as

$${\mathrm{w}}_{1}=\frac{7}{15}\upgamma {\mathrm{w}}_{2}=\frac{7}{15}\upgamma {\mathrm{w}}_{3}=-\frac{14}{15}\upgamma $$

The weight of the clusters can be calculated by Eqs. (50) to (52). In this case, the subjective weights of clusters are:

$${w}_{1}^{s}={w}_{2}^{s}=0.420 {w}_{3}^{s}=0.159$$

The integrated weight of the clusters can be computed by Eq. (53), and when $\mu =0.5$, the weights of clusters are:

$${\mathrm{w}}_{c1}=0.424 {\mathrm{w}}_{c2}=0.373 {\mathrm{w}}_{c3}=0.203$$

The final weights of each expert *w* are shown in Table 9.

**Table 9:** Determined expert weight by the proposed method.

Expert	Weight	Expert	Weight	Expert	Weight
D1	0.0422	M1	0.0351	C1	0.0179
D2	0.0425	M2	0.0389	C2	0.0173
D3	0.0425	M3	0.0380	C3	0.0183
D4	0.0443	M4	0.0370	C4	0.0170
D5	0.0417	M5	0.0388	C5	0.0218
D6	0.0429	M6	0.0367	C6	0.0241
D7	0.0450	M7	0.0358	C7	0.0225
D8	0.0420	M8	0.0366	C8	0.0210
D9	0.0401	M9	0.0392	C9	0.0213
D10	0.0410	M10	0.0368	C10	0.0218

## Comparison of methods with and without using weight determination

Among MADM methods, TOPSIS is widely used in supply chain management, design concept evaluation, business and marketing management, and some other fields. To illustrate the influence of the expert weight, a comparative analysis is made using the TOPSIS method with and without expert weight determination.

The steps of the TOPSIS method are shown here.

***Step 1***: ***Normalize the decision matrix.***

59 $${r}_{ij}={x}_{ij}/\sqrt{\sum {x}_{ij}^{2}}$$

For a decision matrix $$\mathrm{X}=\left\{{x}_{ij}\right\}$$ has $$m$$ alternatives and $$n$$ criteria,$$1\le i\le m$$ and $$1\le j\le n$$, respectively.$$ {r}_{ij}$$ represents the normalized matrix.

***Step 2***: ***Compute the matrix with criteria weight.***

60 $${v}_{ij}={w}_{j}{r}_{ij}$$

where ${w}_{j}$ represents the weight of criterion $j$.

***Step 3:*** ***Calculate the positive ideal solutions (PIS)/negative ideal solutions (NIS).***

61 $$PIS:{A}^{+}=\{{v}_{1}^{+},{v}_{2}^{+},\dots ,{v}_{n}^{+}\}$$

62 $$NIS:{A}^{-}=\left\{{v}_{1}^{-},{v}_{2}^{-},\dots ,{v}_{n}^{-}\right\}$$

where $${v}_{j}^{+}=\{\mathit{max}\left({v}_{ij}\right)\;for\; benefit-type\; criterion; \mathit{min}\;\left({v}_{ij}\right)\; for\; cost-type\; criterion\}$$ and $${v}_{j}^{-}=\{\mathit{min}\left({v}_{ij}\right)\;for\; benefit-type\; criterion;\; \mathit{max}\;\left({v}_{ij}\right)\; for\; cost-type\; criterion\}$$.

***Step 4***: ***Compute the separation measures of alternatives.***

Let ${{\varvec{S}}}_{{\varvec{i}}}^{+}$ / ${{\varvec{S}}}_{{\varvec{i}}}^{-}$ be the distance between the alternative and the PIS/NIS.

63 $${S}_{i}^{+}=\sqrt{\sum {({v}_{j}^{+}-{v}_{ij})}^{2}}$$

64 $${S}_{i}^{-}=\sqrt{\sum {({v}_{j}^{-}-{v}_{ij})}^{2}}$$

***Step 5: Calculate the closeness index (CI) value.***

65 $$CI=\frac{{S}^{-}}{{S}^{+}-{S}^{-}}$$

The rank of the alternatives can be determined by comparing $CIs$. The alternative with the highest $CI$ is the best solution.

The criteria weights are determined by the subjective method, shown in Table 10.

**Table 10:** Criteria weight.

Criteria	C_1_	C_2_	C_3_	C_4_	C5	C_6_	C_7_	C_8_	C_9_	C_10_
${w}_{j}$	0.186	0.084	0.082	0.071	0.028	0.054	0.086	0.108	0.121	0.182

According to the TOPSIS method, the relative variates are calculated. ${S}_{i}^{+}$,${S}_{i}^{-}$, $CIs$ of alternatives with and without expert weights are shown in Table 11.

**Table 11:** Values of ${S}_{i}^{+}$,${S}_{i}^{-}$, $CI$ with and without expert weight.

	Decision made without expert weight			Decision made with expert weight		
	${A}_{1}$	${A}_{2}$	${A}_{3}$	${A}_{1}$	${A}_{2}$	${A}_{3}$
${S}_{i}^{+}$	0.0271	0.0120	0.0095	0.0280	0.0114	0.0124
${S}_{i}^{-}$	0.0033	0.0208	0.0241	0.0023	0.0234	0.0235
$CI$	0.1086	0.6336	0.7169	0.0752	0.6724	0.6541
Rank	3	2	1	3	1	2

**Figure 8:**
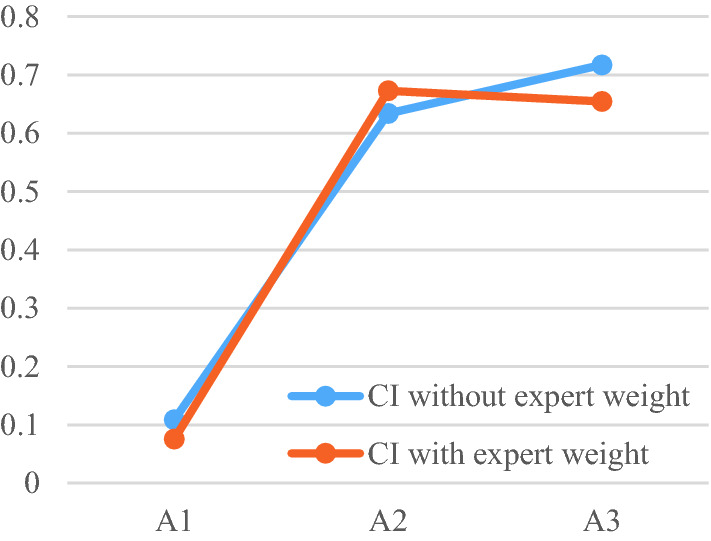
CIs of alternatives with and without expert weight.

From Table 11 and Fig. 8, it is clear that the ranks of alternatives are different with and without considering expert weight. In Fig. 8, both methods demonstrate alternative ${A}_{1}$ is the least ideal option, and far inferior to the other two solutions. However, when the expert weight is considered, the most feasible alternative changed from ${A}_{3}$ to ${A}_{2}$. On the other hand, the variation of ${S}_{i}^{+}$,${S}_{i}^{-}$, $CI$ with and without expert weight is obvious from Table 11.

The difference is caused by the weight of experts. We can infer from the comparative analysis that if the gap between alternatives is huge, the expert weight determination may not influence the final decision. Otherwise, if a gap between alternatives exists but is not particularly large, the expert weight determination may be considered more in decision making. Ignoring the expert weight may cause us to miss the best alternative in real-life projects as verified in this section.

## Conclusions

Expert weight determination in design concept evaluation is a critical part that is usually ignored by decision makers. A proper weight determination can make the decision making process more accurate. This paper presented an integrated two-layer expert weight determination method under a complex design concept evaluation process. In some complex problems, experts are divided into clusters by certain characteristics, and the weight of experts can be calculated by individuals (layer 1) and clusters (layer 2). In the first layer, the minimized variance model is presented to determine the individual weight in each group. In the second layer, a hybrid weight determination method is proposed by combining the entropy weight method and the AHP method. A case study in cruise ship cabin design was implemented using the proposed method. Comparison of the results showed that weight determination in a complex product design process is essential, and may sometimes cause different outcomes.

## Supplementary Information

Supplementary Information.
