# Supplementary material for: An integrated expert weight determination method for design concept evaluation
Source: Sci Rep. 2022 Apr 15;12:6358. doi: 10.1038/s41598-022-10333-6 (PMC9012764; doi:10.1038/s41598-022-10333-6)
Supplement: Supplementary file 1 — Supplementary Information. [file 41598_2022_10333_MOESM1_ESM.docx]

Appendix 1: The raw evaluation data of experts

|  | **Alternative1** | | | | | | | | | | **Alternative2** | | | | | | | | | | | **Alternative3** | | | | | | | | | |
| --- | --- | --- | --- | --- | --- | --- | --- | --- | --- | --- | --- | --- | --- | --- | --- | --- | --- | --- | --- | --- | --- | --- | --- | --- | --- | --- | --- | --- | --- | --- | --- |
|  | **C1** | **C2** | **C3** | **C4** | **C5** | **C6** | **C7** | **C8** | **C9** | **C10** | **C1** | **C2** | **C3** | **C4** | **C5** | **C6** | **C7** | **C8** | **C9** | **C10** | **C1** | | **C2** | **C3** | **C4** | **C5** | **C6** | **C7** | **C8** | **C9** | **C10** |
| **D1** | 7 | 4 | 5 | 4 | 4 | 6 | 6 | 7 | 7 | 7 | 7 | 7 | 7 | 6 | 5 | 5 | 7 | 7 | 8 | 8 | 7 | | 8 | 7 | 7 | 7 | 7 | 6 | 7 | 6 | 8 |
| **D2** | 6 | 4 | 4 | 4 | 5 | 5 | 7 | 7 | 7 | 8 | 8 | 7 | 6 | 7 | 7 | 4 | 8 | 7 | 7 | 7 | 6 | | 7 | 7 | 7 | 7 | 6 | 6 | 8 | 7 | 7 |
| **D3** | 6 | 3 | 4 | 4 | 7 | 5 | 6 | 7 | 8 | 7 | 8 | 5 | 7 | 8 | 7 | 7 | 7 | 7 | 6 | 7 | 6 | | 7 | 7 | 7 | 7 | 7 | 6 | 7 | 6 | 7 |
| **D4** | 5 | 5 | 4 | 4 | 5 | 6 | 6 | 5 | 8 | 7 | 8 | 6 | 7 | 7 | 7 | 4 | 7 | 7 | 7 | 7 | 5 | | 7 | 7 | 8 | 7 | 5 | 7 | 7 | 7 | 7 |
| **D5** | 6 | 5 | 6 | 4 | 5 | 6 | 6 | 5 | 8 | 7 | 7 | 7 | 6 | 7 | 7 | 5 | 7 | 8 | 7 | 7 | 6 | | 7 | 7 | 7 | 8 | 7 | 6 | 8 | 7 | 7 |
| **D6** | 4 | 4 | 6 | 4 | 5 | 6 | 7 | 5 | 7 | 8 | 8 | 7 | 7 | 7 | 4 | 7 | 7 | 8 | 7 | 8 | 4 | | 7 | 5 | 8 | 7 | 7 | 6 | 7 | 8 | 6 |
| **D7** | 6 | 5 | 4 | 5 | 5 | 5 | 6 | 7 | 6 | 8 | 7 | 7 | 6 | 6 | 5 | 5 | 7 | 8 | 7 | 7 | 6 | | 7 | 5 | 8 | 8 | 7 | 5 | 6 | 7 | 7 |
| **D8** | 7 | 5 | 4 | 4 | 5 | 5 | 6 | 7 | 6 | 8 | 7 | 8 | 7 | 8 | 6 | 7 | 6 | 8 | 6 | 7 | 7 | | 8 | 5 | 7 | 8 | 5 | 6 | 6 | 8 | 7 |
| **D9** | 5 | 5 | 6 | 5 | 5 | 6 | 6 | 7 | 6 | 7 | 8 | 7 | 6 | 7 | 7 | 7 | 7 | 8 | 6 | 7 | 5 | | 8 | 7 | 7 | 8 | 7 | 7 | 8 | 8 | 7 |
| **D10** | 6 | 5 | 5 | 5 | 5 | 6 | 6 | 8 | 6 | 7 | 8 | 5 | 7 | 8 | 5 | 5 | 7 | 7 | 8 | 7 | 6 | | 8 | 7 | 7 | 8 | 7 | 6 | 8 | 8 | 6 |
|  |  |  |  |  |  |  |  |  |  |  |  |  |  |  |  |  |  |  |  |  |  | |  |  |  |  |  |  |  |  |  |
| **M1** | 7 | 4 | 5 | 6 | 5 | 7 | 5 | 8 | 7 | 6 | 7 | 7 | 6 | 7 | 6 | 6 | 6 | 6 | 7 | 7 | 7 | | 7 | 6 | 7 | 5 | 6 | 7 | 7 | 7 | 7 |
| **M2** | 6 | 4 | 6 | 6 | 4 | 5 | 5 | 7 | 7 | 6 | 6 | 7 | 6 | 5 | 7 | 7 | 6 | 4 | 7 | 7 | 7 | | 7 | 5 | 6 | 6 | 6 | 7 | 7 | 6 | 6 |
| **M3** | 5 | 4 | 6 | 5 | 5 | 6 | 4 | 7 | 5 | 6 | 7 | 7 | 6 | 7 | 7 | 7 | 6 | 5 | 7 | 7 | 6 | | 7 | 6 | 7 | 5 | 6 | 7 | 7 | 7 | 6 |
| **M4** | 6 | 5 | 7 | 7 | 5 | 6 | 5 | 7 | 7 | 6 | 6 | 7 | 7 | 7 | 7 | 7 | 6 | 5 | 6 | 7 | 7 | | 6 | 5 | 6 | 5 | 5 | 5 | 6 | 8 | 7 |
| **M5** | 6 | 4 | 5 | 5 | 5 | 5 | 7 | 6 | 7 | 7 | 7 | 7 | 6 | 7 | 5 | 6 | 6 | 5 | 6 | 5 | 8 | | 7 | 4 | 6 | 6 | 7 | 7 | 6 | 7 | 6 |
| **M6** | 7 | 5 | 7 | 6 | 4 | 6 | 5 | 7 | 7 | 6 | 5 | 7 | 7 | 7 | 7 | 7 | 6 | 7 | 6 | 7 | 7 | | 6 | 5 | 6 | 6 | 6 | 6 | 6 | 7 | 6 |
| **M7** | 6 | 5 | 6 | 6 | 4 | 6 | 5 | 7 | 6 | 6 | 6 | 8 | 7 | 7 | 7 | 7 | 6 | 5 | 7 | 7 | 7 | | 6 | 6 | 7 | 6 | 7 | 6 | 8 | 7 | 5 |
| **M8** | 7 | 6 | 5 | 5 | 5 | 6 | 5 | 6 | 7 | 6 | 7 | 7 | 7 | 7 | 7 | 7 | 6 | 5 | 7 | 5 | 7 | | 6 | 5 | 7 | 6 | 6 | 6 | 8 | 7 | 6 |
| **M9** | 7 | 5 | 6 | 5 | 4 | 5 | 5 | 7 | 5 | 7 | 7 | 7 | 7 | 6 | 5 | 6 | 6 | 5 | 7 | 7 | 8 | | 6 | 4 | 5 | 7 | 6 | 6 | 6 | 7 | 6 |
| **M10** | 7 | 7 | 7 | 5 | 5 | 6 | 5 | 6 | 7 | 6 | 7 | 7 | 6 | 6 | 7 | 6 | 6 | 5 | 7 | 6 | 7 | | 6 | 5 | 7 | 6 | 6 | 7 | 6 | 7 | 6 |
|  |  |  |  |  |  |  |  |  |  |  |  |  |  |  |  |  |  |  |  |  |  | |  |  |  |  |  |  |  |  |  |
| **C1** | 6 | 6 | 7 | 7 | 5 | 6 | 8 | 5 | 6 | 7 | 7 | 8 | 8 | 7 | 7 | 8 | 7 | 6 | 8 | 8 | 6 | | 8 | 7 | 8 | 7 | 7 | 6 | 7 | 7 | 6 |
| **C2** | 6 | 5 | 7 | 6 | 6 | 7 | 8 | 8 | 7 | 6 | 6 | 8 | 8 | 8 | 8 | 7 | 6 | 7 | 7 | 8 | 6 | | 8 | 8 | 7 | 6 | 6 | 7 | 8 | 8 | 7 |
| **C3** | 7 | 5 | 5 | 6 | 6 | 7 | 8 | 7 | 7 | 6 | 7 | 6 | 6 | 6 | 6 | 7 | 7 | 7 | 7 | 8 | 7 | | 7 | 8 | 7 | 7 | 8 | 7 | 7 | 8 | 7 |
| **C4** | 8 | 6 | 6 | 8 | 7 | 6 | 6 | 8 | 6 | 7 | 8 | 7 | 6 | 8 | 8 | 6 | 6 | 7 | 7 | 8 | 8 | | 7 | 7 | 7 | 8 | 8 | 7 | 7 | 7 | 7 |
| **C5** | 5 | 6 | 6 | 6 | 6 | 7 | 5 | 6 | 6 | 5 | 7 | 6 | 7 | 7 | 6 | 7 | 6 | 6 | 6 | 7 | 5 | | 5 | 6 | 8 | 6 | 6 | 6 | 7 | 7 | 8 |
| **C6** | 6 | 6 | 5 | 5 | 6 | 5 | 7 | 6 | 6 | 5 | 5 | 6 | 5 | 5 | 6 | 5 | 5 | 6 | 6 | 6 | 6 | | 7 | 6 | 6 | 7 | 7 | 7 | 6 | 7 | 7 |
| **C7** | 7 | 7 | 6 | 5 | 6 | 6 | 7 | 8 | 7 | 6 | 6 | 7 | 7 | 6 | 6 | 7 | 5 | 5 | 6 | 6 | 7 | | 6 | 5 | 5 | 6 | 5 | 7 | 6 | 6 | 5 |
| **C8** | 6 | 6 | 6 | 5 | 7 | 5 | 7 | 5 | 6 | 6 | 6 | 7 | 6 | 7 | 6 | 6 | 5 | 6 | 7 | 7 | 6 | | 8 | 7 | 7 | 8 | 6 | 7 | 5 | 6 | 8 |
| **C9** | 5 | 6 | 5 | 6 | 7 | 6 | 5 | 7 | 5 | 6 | 6 | 6 | 6 | 7 | 7 | 6 | 7 | 5 | 6 | 7 | 5 | | 7 | 7 | 8 | 6 | 7 | 7 | 8 | 7 | 6 |
| **C10** | 4 | 5 | 5 | 6 | 5 | 7 | 5 | 8 | 6 | 5 | 5 | 6 | 6 | 6 | 5 | 8 | 8 | 8 | 6 | 7 | 4 | | 7 | 6 | 8 | 5 | 6 | 7 | 6 | 7 | 8 |

Appendix 2: Pairwise comparative judgements based on Table 1

| **Designer cluster** | | | | **Manufacturer cluster** | | | | **Customer cluster** | | | |
| --- | --- | --- | --- | --- | --- | --- | --- | --- | --- | --- | --- |
|  | **D/M** | **D/C** | **M/C** |  | **D/M** | **D/C** | **M/C** |  | **D/M** | **D/C** | **M/C** |
| **D1** | 2 | 4 | 2 | **M1** | 0 | 2 | 4 | **C1** | 2 | 0 | 0 |
| **D2** | 2 | 4 | 2 | **M2** | 0 | 2 | 0 | **C2** | 0 | -2 | -2 |
| **D3** | 2 | 6 | 2 | **M3** | -2 | 0 | 0 | **C3** | -2 | 0 | -2 |
| **D4** | 0 | 2 | 0 | **M4** | 0 | 2 | 4 | **C4** | 2 | 2 | 0 |
| **D5** | 2 | 4 | 2 | **M5** | -2 | 0 | 0 | **C5** | 0 | 0 | -2 |
| **D6** | 0 | 0 | 0 | **M6** | -2 | 0 | 2 | **C6** | 2 | 2 | 0 |
| **D7** | 0 | 2 | 2 | **M7** | 0 | 2 | 2 | **C7** | 2 | 0 | 0 |
| **D8** | 0 | 4 | 4 | **M8** | 0 | 0 | 2 | **C8** | 0 | -2 | 0 |
| **D9** | 2 | 4 | 2 | **M9** | 0 | 0 | 0 | **C9** | 0 | 2 | 0 |
| **D10** | 0 | 4 | 2 | **M10** | -2 | 2 | 0 | **C10** | -2 | 0 | 0 |
| **Ave** | 1 | 3.4 | 1.8 | **Ave** | -0.8 | 1 | 1.4 | **Ave** | 0.4 | 0.2 | -0.6 |

*D is short for designer, M is short for manufacturer and C is short for customer in Appendix1 and Appendix 2.
